# Evaluating the Effectiveness of a Hybrid Model for Mobilization of Women for Cervical Cancer Screening; A Preliminary Report of a Pilot Project in Lagos, Nigeria. A Case Study of the Blue-Pink Center for Women's Health, Nigeria

**DOI:** 10.1200/GO.22.18000

**Published:** 2022-05-05

**Authors:** Idris Ola, Aishat Shoyinka, Olufunmilayo Ayinuola, Loveth Ariomaghwa

**Affiliations:** ^1^ The Blue-Pink Center for Women's Health, Lagos, Nigeria

## Abstract

Cervical cancer is the second commonest cancer in Nigeria, accounting for 14.8% of all cancer-related deaths in Nigeria. Studies have demonstrated poor knowledge and attitude of women toward cervical cancer, with screening rates as low as 0.7% among Nigerian women. Besides poor awareness and poverty, lack of easy access to cancer education and screening is among the biggest barriers to its effective prevention and control. Our project aimed to increase access to cervical cancer education and screening among rural and suburban women in Lagos, Nigeria.

**METHODS:**

We introduced an integrated approach to mobilize women for cervical cancer screening (using Visual Inspection with Acetic Acid [VIA]) in four local councils in Lagos. This model involved a hybrid of a facility- and community-based provision of screening services. Daily, skilled nurses and community health extension workers actively mobilize community women for screening through a purpose-built screening center and regular community outreaches to mirror organized and opportunistic screening. Extensive community stakeholders engagement was used to mobilize women groups. Women were charged up to $2.92 to identify if putting a price deters willingness to screen and to evaluate the model's sustainability. Project evaluation was conducted by a mixed method of quantitative and qualitative assessments.

**RESULTS:**

Within nine months of the pilot, 13,892 women received cancer education, 1,250 women had VIA, and 181 had cryotherapy. Knowledge about cervical cancer rose from an average of 13.7% in our pre-participation survey to 80% in the post-participation surveys. Increased trust in the screening was the commonest theme in the qualitative survey which increased service uptake.

**CONCLUSION:**

Our preliminary findings show that this hybrid model increased access to, and uptake of, cervical cancer screening, even when a cost was attached. Provision of screening services in an organized and strategic people-oriented manner may yield better and sustained outcomes in resource-poor settings than either opportunistic screening or screening offered only as part of routine hospital services.

